# Cesarean section: More than a maternal health issue

**DOI:** 10.1371/journal.pmed.1003792

**Published:** 2021-10-12

**Authors:** Marleen Temmerman, Abdu Mohiddin

**Affiliations:** Centre of Excellence in Women and Child Health, Medical College, Aga Khan University, Nairobi, Kenya

## Abstract

A cesarean section (CS) can be a lifesaving intervention when medically indicated, but it may also lead to adverse short- and long-term health effects for women and children.

## Abstract 2

Marleen Temmerman and Abdu Mohiddin discuss the accompanying study by Enny Paixao and colleagues on associations between cesarean section birth and child mortality up to age 5.

Therefore, the accompanying research study by Paixao and colleagues published in *PLOS Medicine*, looking at CS and associated child mortality in Brazil, provides further valuable evidence on the balance of benefits and risks [1].

CS rates are rising worldwide: Boerma and colleagues, on the basis of data from 169 countries including 98.4% of the world’s births, estimated that 29.7 million (21.1%) births occurred by CS in 2015, almost double the number of CS births in 2000 (16.0 million, 12.1%) [2]. In a further study investigating CS rates in specific obstetric populations using the Robson system, which classifies all deliveries into one of 10 groups on the basis of 5 parameters: obstetric history, onset of labour, foetal lie, number of neonates, and gestational age, there was an increase of CS across most Robson groups, especially after induction of labour in multiparous women [3].

WHO guidance is clear that CS is essential for those who need it, specifying a recommended rate of 10% to 15% to improve maternal and perinatal outcomes and prevent maternal and neonatal mortality and morbidity [4]. Yet, given the increasing use of CS, particularly without medical indication, a more complete understanding of its health effects on women and children has become crucial. The maternal sequelae of CS are well described, while long-term consequences for child health require more research. There is emerging evidence that babies born by CS have different hormonal, physical, bacterial, and medical exposures and that these exposures can subtly alter neonatal physiology. Short-term risks (within 3 years) of CS can include altered immune development; an increased likelihood of allergy, atopy, and asthma; and reduced intestinal gut microbiome diversity [5]. In a systematic review, CS was found to be a risk factor for respiratory tract infections (pooled odds ratio (OR) = 1.30 for asthma) as well as for obesity (pooled OR = 1.35) in children [6]. In a further study including 327,272 neonates born by vaginal delivery and 55,246 by elective CS investigating neonatal respiratory morbidity in relation to mode of delivery, there was a 95% higher risk in neonates delivered by elective CS than in neonates born by spontaneous vaginal delivery [7]. Further, Alterman and colleagues described a moderately elevated risk of severe lower respiratory tract infections during infancy in infants born by planned CS, as compared to those born vaginally [8]. Infants born by planned or emergency CS may also be at a small increased risk of severe upper respiratory tract infections, with a stronger estimated effect if including the indirect effect arising from planning the cesarean birth for an earlier point in gestation than would have occurred spontaneously [8].

However, the extent to which CS, in particular nonmedically indicated CS, benefits or reduces child survival remains unclear. Therefore, Paixao and colleagues conducted a population-based cohort study in Brazil by linking routine data on live births from 2012 to 2018 and assessing mortality up to 5 years of age [1]. Women with a live birth were classified into a Robson group based on pregnancy and delivery characteristics. The analysis of 17,838,115 live births showed that live births to women with low expected frequencies of CS (Robson groups 1 to 4) had a higher death rate up to age 5 years compared with vaginal deliveries (HR = 1.25, 95% CI: 1.22 to 1.28; *p* < 0.001). This means that CS was associated with a 25% increase in child mortality in infants born via CS in Robson groups with low expected frequencies of CS (i.e., low-risk mothers). In groups with high expected frequencies of CS (i.e., high-risk mothers), mortality rates were lower among infants born via CS, supporting the benefits of clinically indicated CS.

This large study shows how important it is to optimise the use of CS, which is increasingly overused leading to global concern. Underuse of CS leads to maternal and perinatal mortality and morbidity, and yet, conversely, overuse of CS has not shown benefits and can create harm. As the frequency of CS continues to increase, interventions to reduce unnecessary CS are urgently needed. As described by Betrán and colleagues, many factors can affect rates of CS, and these may be associated with women, families, health professionals, and healthcare organisations and systems, being influenced by behavioural, psychosocial, health system, and financial factors [9]. These authors concluded that interventions to reduce overuse of CS must be multicomponent and locally tailored, addressing women’s and health professionals’ concerns, as well as reflecting health system and financial factors [9].

Paixao and colleagues’ study provides evidence that either overuse or underuse of CS is associated with child survival, and the findings will help pregnant women and their providers to make informed decisions as to whether CS is appropriate for them. The authors should be commended for carrying out this big data record linkage study, which paves the way for further analyses to study risk profiles using other available population-level data. At a health policy level, the paper shows the significant challenge to child population health that the sequelae of low-risk CS pose, especially in countries with high CS rates such as Brazil at 56% [10]. This represents an avoidable threat to some of the gains to child mortality and morbidity seen over the past few decades and to the achievement of the UN’s Sustainable Development Goal 3 to ensure health and well-being at all ages [11]. Policymakers and civil society groups should take note and act by implementing the recommendations of the 2018 International Federation of Gynaecology and Obstetrics (FIGO) position paper, calling for “joint actions with health professionals, governmental bodies, women’s groups and the healthcare insurance industry to stop unnecessary caesarean sections” [12].

## Abbreviations

CS: cesarean section
FIGO: International Federation of Gynaecology and Obstetrics
OR: odds ratio
